# Sensory Processing Difficulties Correlate With Disease Severity and Quality of Life Among Children With Migraine

**DOI:** 10.3389/fneur.2019.00448

**Published:** 2019-05-24

**Authors:** Jacob Genizi, Ayelet Halevy, Mitchell Schertz, Khaled Osman, Nurit Assaf, Idan Segal, Isaac Srugo, Aharon Kessel, Batya Engel-Yeger

**Affiliations:** ^1^Pediatric Neurology Unit, Bnai Zion Medical Center, Haifa, Israel; ^2^Department of Pediatrics, Bnai Zion Medical Center, Haifa, Israel; ^3^Bruce and Ruth Rappaport Faculty of Medicine, Technion, Haifa, Israel; ^4^Department of Pediatric Neurology, Petach Tikva, Sackler Faculty of Medicine, Schneider Children's Medical Center, Tel Aviv University, Tel Aviv, Israel; ^5^Child Development and Pediatric Neurology Service, Meuhedet-Northern Region, Haifa, Israel; ^6^Division of Allergy and Clinical Immunology, Bnai Zion Medical Center, Haifa, Israel; ^7^Occupational Therapy Department, University of Haifa, Haifa, Israel

**Keywords:** sensory processing, quality of life, children, headache, migraine

## Abstract

**Introduction:** Headaches are common among children and about 80% of children reporting them. Migraine and tension type headaches are the most common primary headaches in children and the prevalence of migraine is about 8%. Accompanying sensory symptoms are common before, during and after migraine attacks. They may be a part of a wider symptom constellation called sensory processing disorder or difficulties (SPD). This includes both hyper or hypo sensitivity to sensations. However, the literature regarding sensory processing symptoms of children and youth with headaches as well as its interaction with child's emotional aspects and quality of life is scarce.

**Materials and Methods:** One hundred and thirty-four children between the ages of 8 and 12 participated in this study. Fifty-four children (22 boys and 32 girls) with episodic migraine were prospectively recruited from pediatric neurological clinics during the years 2014–2017. The control group included 80 healthy children. Both groups completed a health and demographic questionnaire, headache assessment including Ped-MIDAS, Short Sensory Profile, State-Trait Anxiety Inventory (STAI) for children, and the Pediatric Quality of Life Inventory.

**Results:** Children with migraine showed significantly higher prevalence of sensory processing difficulties and lower quality of life compared to healthy controls. Among children with migraine, sensory processing difficulties significantly correlated with lower quality of life. Headache-related disability and sensory processing difficulties predicted quality of life.

**Conclusion:** The possible relationship between migraine and sensory processing disorder or difficulties stresses the need to screen for sensory processing difficulties among children with migraine and when found—refer to their impacts on children's daily function and quality of life.

## Introduction

Headache is one of the leading chronic conditions of childhood (1) and the most common pain complaint when seeking medical advice (2–4), with evidence for increased incidence of primary headaches in children and adolescents in the last 50 years (5, 6). Headaches begin to emerge during the early years of life, but the disorder usually becomes more evident and frequent from the impact of school life, with a peak around 7 years old (7). The prevalence of migraine increases from 3% in the preschool years to 4–11% by the elementary school years, and up to 8–23% during the high school years. The mean age of onset for migraine is 7 years for boys and 11 years for girls (8, 9).

Children who suffer from chronic headaches were found to have more somatic complaints such as abdominal pain and disordered sleep (4, 10), which can also explain why headaches correlate with a significant reduction in quality of life (11, 12). Aromaa et al. (13) investigated pain experience among children with headaches and found they seemed to play more carefully, compared to their family members, because they were afraid of getting hurt. They also found that increased general pain sensitivity proved to characterize children with headache and their parents (13). Migraine in particularly is associated with increased hypersensitivity to various sensory stimuli: visual, auditory, odor, and somatosensory both before aura and during the headache attack (14).

Sensory processing disorder or difficulties (SPD) is a term used to describe difficulties in processing and modulating sensory information in order to respond appropriately to the situation (15). SPD may result in hyper- or hyposensitivity to sensory input. Individuals who are more sensitive to sensory information than others (16) often perceive sensory events as noxious and stressful (17). They are hyperaroused, and more likely to have depression, anxiety disorders as well as social phobia (18) and avoidant personality disorder (19–21). Dunn's model for sensory processing may provide a possible explanation for the relationship between sensory processing abilities and the behavioral output. Dunn's model outlines the relationship between a person's central neurological thresholds and behavioral response (22, 23). Among individuals with hyposensitivity, the central mechanisms of habituation support high thresholds. On the other hand, among individuals with low thresholds, the neurons trigger more easily and thus, cause more frequent reactions to stimuli from the environment resulting in hypersensitivity (23).

Nevertheless, the knowledge about the ability of children with migraine to process sensory input is limited. Since sensory processing abilities have a direct impact on daily function (24) and quality of life (25), by exploring the prevalence of SPD among children with migraine and their impacts on children's quality of life, intervention programs may be more efficient. Hence, the aims of this study were: (1) Compare sensory processing abilities between children with migraine and healthy controls (2) Compare the quality of life between children with migraine and healthy controls (3) Examine the correlations between sensory processing, migraine characteristics and severity and quality of life among children with migraine (4) Examine the contribution of headache-related disability and sensory processing to the prediction of quality of life among children with migraine.

It was hypothesized that children with migraine will have more difficulties to process sensory information and lower quality of life as compared to healthy controls; that sensory processing would correlate with enhanced migraine pain and with lower quality of life and that Sensory processing difficulties and headache-related disability will significantly predict quality of life.

## Materials and Methods

### Participants

According to G-Power software (26), to identify an effect size of 0.25, with *p* = 0.05 and power of 0.80, a total sample of 92 participants is recommended. Each group should include 46 participants. One hundred and thirty-four children between the ages of 8 and 12 years participated in this study. Sixty children with episodic migraine were prospectively recruited from the following outpatient pediatric neurology clinics: (1)The pediatric neurology clinics at the Bnai- Zion Medical Center, (2) the pediatric neurology clinics at the Schneider Children's Medical Center, Petach Tikva, and (3) the pediatric neurology clinics at the Meuhedet Medical Services in the city of Haifa, during the years 2014–2017. Out of 60 children: 57 agreed to participate in the study and 54 (22 boys and 32 girls) completed the questionnaires. The control group included 80 healthy children, 37 boys and 43 girls, who did not have any significant illnesses; did not have positive neurological findings or developmental disorders. Table 1 summarizes the study and control groups' demographic information (Table 1).

**Table 1:** Participants' health and demographic information.

		**Children with migraine (*n* = 54)**	**Healthy controls (*n* = 80)**
Age range		7–12	7.5–11
Mean age ± SD		10.06 ± 1.53	9.33 ± 1.14
Gender - *n* (%)	Boys	22 (40.7%)	37 (46.3%)
	Girls	32 (59.3%)	43 (53.7%)
MIDAS level (%)	No functional impairments	36.7%	
	Minimal functional impairments	30%	
	Moderate functional impairments	16.7%	
	Severe functional impairments	16.7%	
VAS (range, mean ± SD)	6–10, 8.33 ± 1.43		
**Headache frequency (%)**			
Once a week	43.8		
Twice a month	40.7		
Twice a year	15.5		
Mean ± SD of headache frequency (per month)	2.51 ± 0.84		
Median of headache frequency (per month)	2		
**Duration of episodic migraine (%)**			
1 h	26		
2 h and more	62		
12–24 h	10		
More than 1 day	2		

*SD, standard deviation*.

## Methods

### Medical Assessment

A prospective medical history including a thorough headache history and physical and neurological assessment by a pediatric neurologist, were performed on all children during the visit at the pediatric neurology clinic. All children met the diagnostic criteria for migraine, according to the International Classification of Headache Disorders, 3rd edition (ICHD-3 beta) (27). Allodynia was not formally assessed.

### PedMIDAS

Headache related disability was evaluates by the PedMIDAS questionnaire. It was developed to assess migraine disability in pediatric and adolescent patients and has been tested and validated for ages 4–18 (28).

### The Short Sensory Profile (SSP) (26)

This parent report evaluates children's sensory processing patterns, as expressed in all sensory modalities and in daily living situations (for example: “will only eat certain tastes”; “reacts emotionally or aggressively to touch”). The Parent scores their child's response to sensory stimuli on a 5 point Likert scale, where 1 represents “always” and 5 “never.” Seven subtests are scored: tactile sensitivity, taste/smell sensitivity, sensitivity to movement, visual/auditory sensitivity and auditory filtering, as well as a total score, which ranges from 38 to 190. Higher scores (155–190) reflect typical/normal performance. A score between 142 and 154 reflects a probable difference in performance while a score between 38 and 141 reflects a definite difference in performance (29, 30).

### The Pediatric Quality of Life Inventory (PedsQL) (31)

We used Version 4.0—child's report, which profiles children's Health-Related Quality of Life (HRQoL) in four dimensions: (1) Physical Functioning (eight items), (2) Emotional Functioning (five items), (3) Social Functioning (five items), and (4) School Functioning (five items). A higher order dimension of the Psychosocial Health dimension encompasses emotional and social functioning. The child marks the frequency of problems which occurred in the past 1 month on a five-point Likert scale (0 = never a problem; 1 = almost never a problem; 2 = sometimes a problem; 3 = often a problem; 4 = almost always a problem). Items are then transformed into a 0–100-point scale (0 = 100; 1 = 75; 2 = 50; 3 = 25; 4 = 0) to present the HRQoL percentage. A higher percentage indicates a better HRQoL.

## Procedure

After receiving ethical approval from the Bnai Zion Medical Center Ethics Review Board, children from the study group were recruited during their visit at the neurology clinics as described above. All patients' parents signed an informed consent to participate in the study. The headache history was taken and the neurological examination was performed during the visit. After the diagnosis of migraine was made according to ICHD-3 beta (27), including the PedMIDAS questionnaire, the children's parents were asked to complete the Short Sensory Profile and the Pediatric Quality of Life Inventories. Children from the control group were recruited after their parents answered the advertisements calling to participate in the study by contacting the study conductor, and after having met the inclusion criteria. The controls were evaluated in their homes.

## Data Analysis

Normality tests were applied and most dependent variables showed abnormal distribution. Hence, Mann–Whitney test was used to examine if significant differences existed between both groups in SSP and PedsQL scores. Chi square analysis examined whether significant differences existed between groups in the percentage of children found in each of the SSP performance ranges (typical performance; probable difference in performance and definite difference in performance). Among children with migraine, Spearman correlation examined the correlations between sensory processing patterns, migraine characteristics/related disability and quality of life. Stepwise linear regression was examined to identify the relative contribution of MIDAS and SSP scores to the prediction of HRQoL. The level of significance was adjusted for multiple testing for all analyses using Bonferroni correction.

## Results

### Comparing the Sensory Processing Abilities Between Children With Migraine and Healthy Controls

Children with migraine had lower scores (greater sensory processing difficulties) than healthy controls in SSP total scores and in all SSP subtests. This difference was significant only in regards to taste/smell sensitivity (Table 2).

**Table 2:** Comparing the Short Sensory Profile scores between children with Migraine and healthy controls using Mann–Whitney test.

	**Children with migraine (*****n*** **=** **54)**			**Healthy controls (*****n*** **=** **80)**			
**SSP subtest**	**Median**	**Mean ± SD**	**Range**	**Median**	**Mean ± SD**	**Range**	***Z***
Tactile sensitivity	32	31.13 ± 3.35	21–35	33	32.58 ± 2.36	23–35	−2.66
Taste/smell sensitivity	17	16.12 ± 3.86	5–20	19	18.32 ± 2.07	9–20	−3.45^***^
Movement sensitivity	14	13.31 ± 2.34	5–15	15	14.03 ± 1.63	7–15	−1.97
Under responsive/seek	31	29.98 ± 4.88	17–35	32	31.21 ± 3.41	22–35	−0.91
Auditory filtering	25.5	24.84 ± 4.48	13–30	26	25.73 ± 3.41	10–30	−0.73
Low energy/weak	29	27.27 ± 3.66	5–25	30	28.37 ± 2.35	20–30	−1.92
Visual/auditory sensitivity	23	21.89 ± 3.81	5.00	25	23.82 ± 1.84	16–25	−2.53
Total	169.5	164.58 ± 19.94	102–190	175	174.11 ± 9.35	156–190	3.29

*The level of significance was adjusted to p ≤ 0.006*.

*** *p ≤ 0.001; SD, standard deviation. Lower scores indicate worse sensory processing*.

Based on Chi square analysis, significantly higher percentage of children with migraine was found in the definite difference performance range in the taste/smell sensitivity and in the SSP total score (Table 3), representing sensory processing difficulties expressed in hypersensitivity.

**Table 3:** Comparing differences between groups in the percentage of children found in each of the SSP performance range using Chi square analysis.

	**Typical performance**		**Probable difference**		**Definite difference**		
**SSP subtest**	**Migraine**	**controls**	**Migraine**	**controls**	**Migraine**	**controls**	**χ^2^**
Tactile sensitivity	36	64	61.5	38.5	71.4	28.6	6.15
Taste/smell sensitivity	33	67	81.8	18.2	87.5	12.5	17.81^***^
Movement sensitivity	35.2	64.8	55.6	44.4	63.6	36.4	5.35
Underresponsive/seek	35.8	64.2	50	50	77.8	22.2	6.81
Auditory filtering	35.8	64.2	50	50	70	30	5.24
Low energy/weak	36.1	63.9	60	40	54.5	45.5	4.13
Visual/auditory sensitivity	35.8	64.2	85.7	14.3	100	0	12.97
Total SSP	32.8	67.2	100	0	100	0	25.02^***^

*The level of significance was adjusted to p ≤ 0.006*.

*** *p ≤ 0.001*.

### Comparing the HRQoL Between Children With Migraine and Healthy Controls

Children with migraine reported lower Health-Related Quality of Life than healthy controls. However, this difference was significant only in the physical domain (Table 4).

**Table 4:** Comparing the HRQoL between children with Migraine and healthy controls using Mann–Whitney test.

	**Children with migraine** **(*n* = 54)**		**Healthy controls** **(*n* = 80)**		
	**Median**	**Mean ± SD**	**Median**	**Mean ± SD**	***Z***
Physical HRQOL	84.37	81.97 ± 13.44	90.62	89.08 ± 11.37	−3.25^***^
Emotional HRQOL	70	69.91 ± 18.56	75	74.31 ± 15.44	−1.21
Social HRQOL	95	88.21 ± 13.85	95	90.06 ± 13.44	−0.77
School HRQOL	75	74.23 ± 16.09	80	79.81 ± 14.32	−1.86
Psychosocial HRQoL	78.33	77.31 ± 12.85	81.66	81.39 ± 10.01	−1.64
Total HRQoL	81.33	78.26 ± 12.13	83.18	82.93 ± 9.47	−2.01

*The level of significance was adjusted to p ≤ 0.008*.

*** *p ≤ 0.001; SD, standard deviation. Higher scores indicate better HRQoL*.

### The Correlations Between Sensory Processing, Migraine Characteristics/Related Disability, and Quality of Life Among the Study Group

After performing Bonferronni correction, (*p* ≤ 0.004), no significant correlations were between sensory processing and migraine characteristics/related disability. However, lower physical HRQOL significantly correlated with greater movement sensitivity and lower energy. Lower emotional HRQOL significantly correlated with greater tactile sensitivity, visual/auditory sensitivity. Lower emotional and school HRQOL significantly correlated with more extreme sensory processing patterns as represented by the total SSP score. Most correlations were found between psychosocial HRQOL and SSP scores: lower psychosocial HRQOL significantly correlated with greater sensitivity to taste/smell, movement, auditory filtering, low energy, with more extreme sensory processing patterns represented by the total SSP score. Table 5 summarizes the correlations.

**Table 5:** The correlations between sensory processing patterns and quality of life among children with Migraine using Spearman correlation test.

	**Physical HRQOL**	**Emotional HRQOL**	**Social HRQOL**	**School HRQOL**	**Psychosocial HRQOL**	**Total**
Tactile sensitivity	0.34	0.43^***^	0.11	0.19	0.37	0.39
Taste/smell sensitivity	0.42	0.41	0.24	0.324	0.47^***^	0.48^***^
Movement sensitivity	0.49^***^	0.42	0.23	0.362	0.49^***^	0.54^***^
Under responsive/seek	0.28	0.38	0.17	0.293	0.39	0.42
Auditory filtering	0.31	0.44	0.34	0.426	0.54^***^	0.55^***^
Low energy/weak	0.53^***^	0.31	0.38	0.424	0.46^***^	0.54^***^
Visual/auditory sensitivity	0.16	0.44^***^	0.11	0.320	0.42	0.41
Total	0.45^***^	0.55^***^	0.31	0.44^***^	0.61^***^	0.63^***^

*The level of significance was adjusted to p ≤ 0.004*.

*** *p ≤ 0.001*.

### Predicting the Quality of Life Children With Migraine by Their Measure Headache-Related Disability (PedMIDAS Score) and Sensory Processing

After adjusting the level of significance to *p* ≤ 0.01, stepwise linear regression analysis revealed that emotional HRQOL was significantly predicted by tactile sensitivity. accounting for 22% of the variance [F_(1, 28)_ = 8.29; B = 2.86; SE B = 0.99; β = 0.47, *p* ≤ 0.01]. Social HRQOL was significantly predicted by PedMIDAS score, accounting for 25% of the variance [F_(1, 28)_ = 9.71; B = −0. 32; SE B = 0.11; β = −0.51, *p* ≤ 0.01].

## Discussion

The main outcomes of the present study found that sensory processing difficulties are prevalent among children with migraine and that their quality of life is predicted by both headache-related disability and sensory processing difficulties.

A connection between migraine and sensory processing difficulties is not surprising. Patients with migraine tend to have enhanced perception of various sensory stimuli including sound, somatosensory stimuli (14) odors (32, 33), and increased sensitivity to light during and between migraine attacks (34). According to some reports, smells and flashing lights are triggers of migraine attacks. These symptoms correlate with the findings that have atypical symmetry and amplitude of the initial negative and positive cortical responses to visual stimuli (35) and different high frequency oscillations of the somatosensory evoked potential compared to controls (36). Another finding, irrespective of the stimulus modality, is an impairment of habituation in interictal migraineurs as compared to healthy controls (37). Enhanced sensory sensitivity and habituation difficulties among patients with migraine were also observed in studies that applied quantitative sensory testing (QST) (38) noting that patients with migraine may have greater reactivity to pain. The meta analysis performed by Nahman-Averbuch et al. (39) revealed that patients with migraine present lower heat and pressure pain thresholds, higher pain ratings to cold suprathreshold stimuli for combined and nonlocal areas, and higher pain ratings to electrical suprathreshold stimuli for nonlocal areas, than healthy controls. All these findings raise the hypothesis that migraineurs might have basal abnormalities in sensory processing and integration. Tyll and Noseda both (40, 41) suggested that sensory hypersensitivity may result from activation of subcortical brain regions that receive convergent inputs and then project broadly to various cortical brain regions involved in integrating multiple sensory modalities such as visual, auditory, and olfactory. Mainero et al. (42) demonstrated that patients with migraine have stronger connectivity between the ventrolateral periaqueductal gray (PAG) and other brain areas that are involved in nociceptive and somatosensory processing. Recently it has been proposed (34, 43) that both the aura and the migraine attack, may represent a form of hypersensitivity due to sensory processing difficulties.

The present study used the Short Sensory Profile in order to measure sensory processing abilities, as reflected in children's daily life. In the present study, a relatively high percentage of children with migraine were found to score in the “definite difference” range on most SSP scales.

The other main outcome of the present study was that children with migraine had lower quality of life in various domains as compared to healthy controls. This is supported by previous reports. For example, Powers (11, 12) found that migraine may reduce children's QoL, and this impact may differ by age group: teens reported lower school functioning than older and younger children and younger children reported lower social functioning than older children and teens (11). Physical complaints as well as mental problems can adversely affect a patient's quality of life (QOL) (44, 45). This may be reflected directly by children's self-reports, as found in our study.

The present study is the first, to our knowledge, to find a correlation between the reduction in social quality of life in children with migraine and the PedMIDAS score. Nevertheless, this study not only supports the relationship between migraine influence and children's HRQoL, but it brings innovative information about the involvement and contribution of sensory processing difficulties to the prediction of children's HRQoL. This prediction together with the result according to which greater sensory processing difficulties correlated with lower quality of life in the physical as well as in the psychosocial and school domains, emphasizes the relevance of screening for sensory processing difficulties among children with migraine and refer to their impacts on child's daily life in intervention programs.

Moreover, based on previous reports highlighting the correlations between sensory processing difficulties, emotional status and hyperarousability (that frequently characterize individuals with migraine), intervention programs should consider the commonality of anxiety disorders, depressive disorders and other forms of psychopathology in children, and adolescence with migraine (4, 46–48) with respect to sensory processing difficulties and to quality of life. By referring to these interactions in research and practice, we may better understand other factors, such as SPD, that may be associated with higher levels of somatic and emotional complaints in children that lead to poorer school attendance, school refusal, and poorer academic performance (49, 50). Thus, by applying this broad perspective screen for SPD, early intervention may be provided, focusing on providing coping strategies to deal with the sensory difficulties and optimize function. By that, clinicians may reduce the negative consequences of migraine and related difficulties in terms of social, academic and personal adjustment (51, 52), and elevate children's HRQoL.

## Limitations

Our study has a few limitations. This study was conducted in tertiary pediatric clinics, and not on a sample of healthy children (like a school-based study). According to Berkson's principal (53), people who seek medical care are more likely to have more than one medical problem. Therefore, the relationship between two diseases should not be studied in such a population. In addition, in this study we did not formally assess allodynia.

## Conclusions

Sensory processing difficulties may characterize children with migraine and reduce their quality of life. Hence, sensory processing difficulties should be screened and treated when relevant, with respect to their impacts on children's daily function and quality of life. The implication of these findings as regards the treatment of migraine in children needs further study.

## Ethics Statement

This study was carried out in accordance with the recommendations of name of guidelines, Bnai Zion IRB number bnz 21-14 with written informed consent from all subjects. All subjects gave written informed consent in accordance with the Declaration of Helsinki. The protocol was approved by the Bnai Zion IRB number bnz 21-14.

## Author Contributions

All authors listed have made a substantial, direct and intellectual contribution to the work, and approved it for publication.

### Conflict of Interest Statement

The authors declare that the research was conducted in the absence of any commercial or financial relationships that could be construed as a potential conflict of interest.
